# Role of human epidermal growth factor receptor 3 in treatment resistance of anaplastic lymphoma kinase translocated non-small cell lung cancer

**DOI:** 10.1080/15384047.2023.2256906

**Published:** 2023-09-18

**Authors:** Tiia J Honkanen, Milla E K Luukkainen, Jussi P Koivunen

**Affiliations:** a Department of Oncology and Radiotherapy, Oulu University Hospital, Oulu, Finland; b Medical Research Center Oulu, Oulu, Finland; c Cancer and Translational Medicine Research Unit, University of Oulu, Oulu, Finland

**Keywords:** HER3, ALK, NSCLC, treatment resistance, interaction

## Abstract

**Background:**

ALK tyrosine kinase inhibitors (TKI) have revolutionized the treatment of *ALK*+ non-small cell lung cancer (NSCLC), and therapy resistance occurs in virtually all patients. Multiple TKI resistance mechanisms have been characterized, including ERBB receptor coactivation. In this study, we investigated the role of HER3 in ALK TKI resistance.

**Methods:**

*In vitro* studies were carried out using *ALK*+ NSCLC cell lines H3122, H2228, and DFCI032. Pharmacological co-targeting of ALK and HER3 was investigated with ALK and ERBB TKIs, and HER3 knockdown was achieved using the CRISPR-Cas9 system. Co-localization of ALK and HER3 was investigated by immunoprecipitation (IP) and proximity ligation assay (PLA) *in vitro* and *in vivo* using six *ALK+* NSCLC tumor samples.

**Results:**

In all tested cell lines, combined targeting with ALK and pan-ERBB TKI resulted in marked inhibition of colony formation and long-term (72 h) downregulation of pAKT levels. HER3 knockdown resulted in multiple effects on ALK+ cell lines, including the downregulation of ALK expression and visible morphological changes (H2228). Co-immunoprecipitation (IP) and proximation ligation assay (PLA) experiments provided evidence that both ALK and HER3 could interact *in vitro*, and this finding was verified by PLA using *ALK*+ NSCLC tumors.

**Conclusions:**

This study provides evidence that HER3 may mediate TKI resistance in *ALK*+ NSCLC. Interestingly, we were able to show that both translocated ALK and HER3 could interact. Joint targeting of ALK and HER3 could be further investigate in *ALK*+ NSCLC.

## Introduction

Approximately 3–7% of non-small cell lung cancers (NSCLC) contain chromosomal rearrangements of anaplastic lymphoma kinase (ALK), resulting in constitutively active ALK.^1,2^ ALK rearranged NSCLCs are highly sensitive to ALK tyrosine kinase inhibitors (TKIs), such as crizotinib and alectinib, and intrinsic TKI resistance is rare. Acquired resistance to ALK TKIs is inevitable. Various ALK TKI on- and off-target resistance mechanisms have been reported, such as secondary mutations in *ALK* and activation of bypass signaling pathways, though other tyrosine kinase receptors such as EGFR.^3,4^

Human epidermal growth factor receptor (HER/ERBB) family consists of four members: EGFR/HER1, HER2, HER3 and HER4. When these receptors are activated, they form homo- or heterodimers and signal through pathways that are essential for cell proliferation and survival, such as the MAPK and PI3K-pathways.^5,6^ EGFR and HER2 are commonly altered in cancers, including non-small cell lung (NSCLC) and breast cancers, whereas oncogenic activation of HER3 and HER4 is uncommon.^7–10^ Although the kinase domain of HER3 is defective, it is an important dimerization partner of EGFR and HER2, and HER2 amplified cancers have been shown to depend on HER3 for downstream signaling to PI3K.^11,12^ Furthermore, neuregulin-1, a HER3 and HER4 ligand, has been shown to induce epithelial–mesenchymal transition and expression of other proteins involved in invasion and metastasis and to drive therapy resistance.^13–16^

Secondary *ALK* mutations have been reported to mediate the acquired resistance to ALK TKIs. Common acquired resistance mutations for crizotinib are L1196M and G1269A, whereas alectinib and lorlatinib have wider coverage for acquired mutations. Multiple ALK TKI off-target resistance mechanisms have been characterized such as activation of ERBB-family, c-MET, c-KIT, IGF-1 R-IRS-1, and MAPK pathways.^4,13,17–19^ Of the HER-family members, EGFR, HER2, and HER3 have previously been linked to ALK TKI resistance by activation of bypass signaling.^20^ In *ALK+* cancers, HER3 activation can occur through heterodimer formation or by cytokine NRG1.^21^ Furthermore, co-targeting of ALK and HER3 has been shown to overcome ALK TKI resistance.^22,23^ Currently, there are no approved therapies for off-target ALK TKI resistance and compared to on-target resistance, less is known about the phenomenon. As drug resistance develops in virtually all patients, studying the molecular mechanisms and novel treatment approaches is warranted.

We have previously investigated the role of HER2 in ALK TKI resistance. Our previous results suggested that HER2-HER3 heterodimers would play a role in TKI resistance.^24^ Therefore, we wanted to further investigate the significance of HER3 in *ALK* translocated NSCLC. We speculate that HER3 and ALK co-targeting could prove to be a novel treatment approach for ALK TKI resistance.

## Materials and Methods

### Cell lines, tumors, and reagents

ALK-translocated NSCLC cell lines were received as a kind gift from Dr Pasi Jänne (DFCI, Boston, MA, USA) and their characteristics, sensitivity, and resistance to ALK TKIs is described in a previous publication.^20^ All the cell lines used were the primary and none of them were made resistant *in vitro* by long-term exposure to TKIs. Furthermore, ALK tyrosine kinase sections of all of these cell lines have been sequenced and they bear no secondary mutations. H3122 and H2228 cell lines were cultured in RPMI-1640 medium (SH30027.01, Cytiva, USA) with added 10% FBS (fetal bovine serum, S-FBS-SA-015, Serana, Germany) and 1% penicillin and streptomycin (SV3001.01, Cytiva, USA). DFCI032 line was cultured in ALC-4 medium with 10% FBS and 1% p/s and DMEM/F-12 (1:1) with HEPES (31330–038, Gibco, USA) as the base medium. All cultured cells were placed in incubator with 5% carbon dioxide at +37°C. 0.25% Trypsin (SH30042.01, Cytiva, USA) was used to detach cells for harvesting. All cell lines tested negative Mycoplasma. TKI drugs used in the experiments were crizotinib (1 µM), afatinib (1 µM), and lapatinib (1 µM). The drugs were purchased from LC Laboratories (Woburn, MA, USA). Treatment drugs were dissolved in DMSO and stored at −20°C in aliquots to minimize thawing cycles. The specific exposure times to TKIs were selected based on experimental goals with short (2–5 h; initial changes in phosphorylation and co-localization) and long-term (24 h-7 d; cell survival and resistance mechanisms) exposures and preliminary pilot experiments.

ALK-translocated NSCLC FFPE tumor samples (*n* = 6) were obtained from Auria Biobank (Turku, Finland) with permission number BB_2020–0035.

### Colony formation assay

Into a flat bottomed 24-well plate, a total of 800–1000 cells were seeded and let to attach to the bottom surface for 24 h before treatment. Treatments were performed in two parallel, and untreated cells were used as controls. This experiment was repeated multiple times to obtain consistent results. Drug incubation was carried out for 7 days, and the fresh medium was changed for free cell proliferation. Cells were allowed to proliferate until changes appeared, and fresh medium was changed during the proliferation period when needed. After notable change, cells were then washed with PBS, fixed with ice-cold methanol, and stained with 0.005% crystal violet (Merck, Darmstadt, Germany).

### Western blot

*A* total of 5000–8000 cells per well were seeded in flat bottomed 6 well plates and allowed to attach for 1–2 days. After the drug treatment, cells were washed in PBS and lysed with NP-40 lysis buffer (1% Igepal CA-630, 20 mM Tris – HCl pH 8.0, 137 mM NaCl, 10% glycerol, 2 mM EDTA, 1 mM sodium orthovanadate, 10 lg/ml aprotinin, and 10 lg/ml leupeptin). Protein concentrations of the lysates were measured using a Bio-Rad Protein Assay (Bio-Rad, Hercules, CA, USA), and absorbance was measured at 595 nm. The samples were diluted and equalized to the lowest concentrations using distilled water. 3X sample buffer was then added to each sample, and the samples were boiled for 5 min and stored at −80°C. Equal volumes of samples were loaded on a 7,5% SDS-PAGE gel, and separated by electrophoresis, and transferred to a PVDF membrane. The Blocking against nonspecific antibody binding was performed by incubating the membranes in 5% BSA (in PBS with 0.1% Tween-20 and 0.0025% sodium azide) and then incubated in the primary antibodies (1:1000) in 5% BSA-PBS. The following primary antibodies were purchased from Cell Signaling Technology (Danvers, MA, USA): ALK #3633, phospho-ALK #3341, GAPDH #5174, phospho-ERK ½ #9101, phospho-AKT #4060, ERK ½ #1902, AKT #4691, phospho-HER3 #2842, HER3 # 12708, EGRF#4267, pEGRF #3777, HER2 #2165, phospho-HER2 #2243, HER4 #4795, phospho-HER4 #4757, and β-actin from Novus (NB600–501, St. Charles, MO, USA) overnight at +4°C. The membranes were washed with PBS-T the next day, incubated with horseradish peroxidase (HRP)-linked secondary antibodies, and then washed again with PBS-T. All washings and incubations were performed on a low-speed horizontal shaker. The membranes were developed using an Immobilon Western Chemiluminescent HRP Substrate kit (Millipore; Billerica, MA, USA), and the signals were detected on radiographic films. All western blot experiments were performed in duplicate.

### Flowcytometry

For the analysis, 2 million cells per treatment were seeded onto a 10 cm culture dish. After 48 h, the cells were detached using Accutase™ (BD Biosciences, Franklin Lakes, NJ #561527). Cells were then washed with PBS and resuspended in FACS buffer (0.5% BSA +2 mM EDTA in PBS). For HER3 analysis, 5 µL of PE-HER3 (clone 1B4C3) antibody (BioLegend #324705, RRDI: AB_756159) was added to the samples and control treatment, and 5 µL of FACS buffer was added to the controls for 30 min incubation in the dark. The samples were then washed twice with FACS buffer before FACS analysis. FACS analysis was performed using Accuri™ and the results were analyzed with FlowJo™ v10.8 Software (BD Life Sciences).

### Immunoprecipitation

A total of 1.5–3 million cells were seeded into 10 cm culture dishes and allowed to proliferate up to 80–90% confluency. For non-control cells, a 2-h ALK-TKI treatment was conducted before harvesting. Cells were then washed in cold PBS on ice and lysed using NP-40 lysis buffer (1% Igepal CA-630, 20 mM Tris – HCl pH 8.0, 137 mM NaCl, 10% glycerol, 2 mM EDTA, 1 mM sodium orthovanadate, 10 µl [l g/ml] aprotinin and 10 µl [l g/ml]) leupeptin by incubating dishes on ice for 30 min. Samples were then transferred to 1.5 ml Eppendorf tubes, and debris was removed by centrifuging the tubes at 4°C. Protein concentrations of the samples were measured with Bio-Rad Protein Assay (Bio-Rad, Hercules, CA, USA) in the same way as for western blotting. Immunoprecipitation samples (200 µL in 1 µg/µL) were prepared by diluting the lysates with sterilized and autoclaved water. Primary antibodies were then added to the suspensions (ALK 1:100 #3633, HER3 1:50 #2243, and GAPDH 1:200 #5174, Cell Signaling, USA), and the samples were incubated in rotation overnight at 4°C. Protein A/G PLUS-agarose beads (sc-2003, Santa Cruz Biotechnology, Dallas, TX, USA) were added to the samples and incubated in rotation for 3 h. Samples were then centrifuged at 4°C, and the supernatant was discarded. Samples were then washed on four separate occasions by centrifuging it as before at +4°C and by always adding new cold PBS after removing the old one. The pellet was then resuspended in 3X sample buffer, mixed by vortexing, spun down, boiled for 5 min, centrifuged again, and the supernatant was transferred to a new cold Eppendorf. Western blotting was performed as described above.

### Proximity ligation assay (PLA)

In the cell-line experiments, 8000 cells were seeded on 8-chamber glass slides, with five chambers per cell line. Cells were allowed to attach to the glass slides for 2 h before carefully adding 500 µL of the growing medium. The cells were washed with PBS after 24 h and fixed in 4% PFA for 15 min. For ALK TKI treatment, cells were treated with ALK TKIs for 2 h before fixation. PLA was also performed on FFPE tumor samples. Samples were left at +37°C overnight to soften the paraffin, and next morning, de-paraffinization and rehydration were performed on 3 × 5 min Histoclear and brief soaking in AbsEtOH/Histoclear 1:1 solution, followed by 2 × 5 min EtOH to 2 × 5 min absEtOH to 96% and brief soaking in 70% EtOH. Epitope retrieval was performed by microwaving the samples in a Tris-EDTA (pH 9) solution for 10 min. After samples had cooled down while kept on retrieval solution, permeabilization was performed for 10 min with 0.2% Triton X solution in PBS. The cell-line samples were permeabilized with Triton-X solution as tumor samples.

A proximity ligation assay (PLA) was performed using a Duolink® PLA Fluorescence kit (cat. DUO92101-1KT; Merck, Darmstadt, Germany) according to the manufacturer’s instructions. In brief, primary antibodies (mouse HER3 1:50, sc-415, Santa Cruz Biotechnologies, Dallas, TX, USA, and rabbit ALK 1:100 #3633, Cell Signaling, Danvers, MA, USA) were incubated at 4°C overnight. The probe incubation, ligation, and amplification were performed at 37°C. All incubations were performed in a humidity chamber, as instructed by the manufacturer. After exposing the samples to mounting medium, the covering glass was sealed with clear nail polish and samples were visualized with a confocal microscope (Leica SP8 FALCON confocal microscope) with a 40 × or 63 × objective.

### In silico analysis in cBioportal and image analysis

*In silico* analysis of HER3 expression in ALK-translocated NSCLC was performed using cBioPortal data (https://www.cbioportal.org/). mRNA and protein expression levels were compared between wild-type lung adenocarcinomas and ALK-translocated NSCLC.^25,26^ Colony formation image analysis was performed on the original ImageJ1.45 program^27^ the threshold method and particle analysis to obtain values. For fluorescent-positive PLA signals, the color threshold method was used to obtain the signal counts, and cell counts were performed manually. For the tumor samples, positive cell areas were manually counted. Bar charts were created using GraphPad Prism 5.04 and overall image constructs were constructed using Adobe Photoshop CC 2017.

### Statistics

Statistical calculations were performed using GraphPad Prism 5.04 and/or SPSS 25.0.0. For the comparisons, Student’s two-tailed t-test was applied with 95% confidence interval. Statistical significance was set at *P* ≤ .05.

## Results

**Figure 1.**
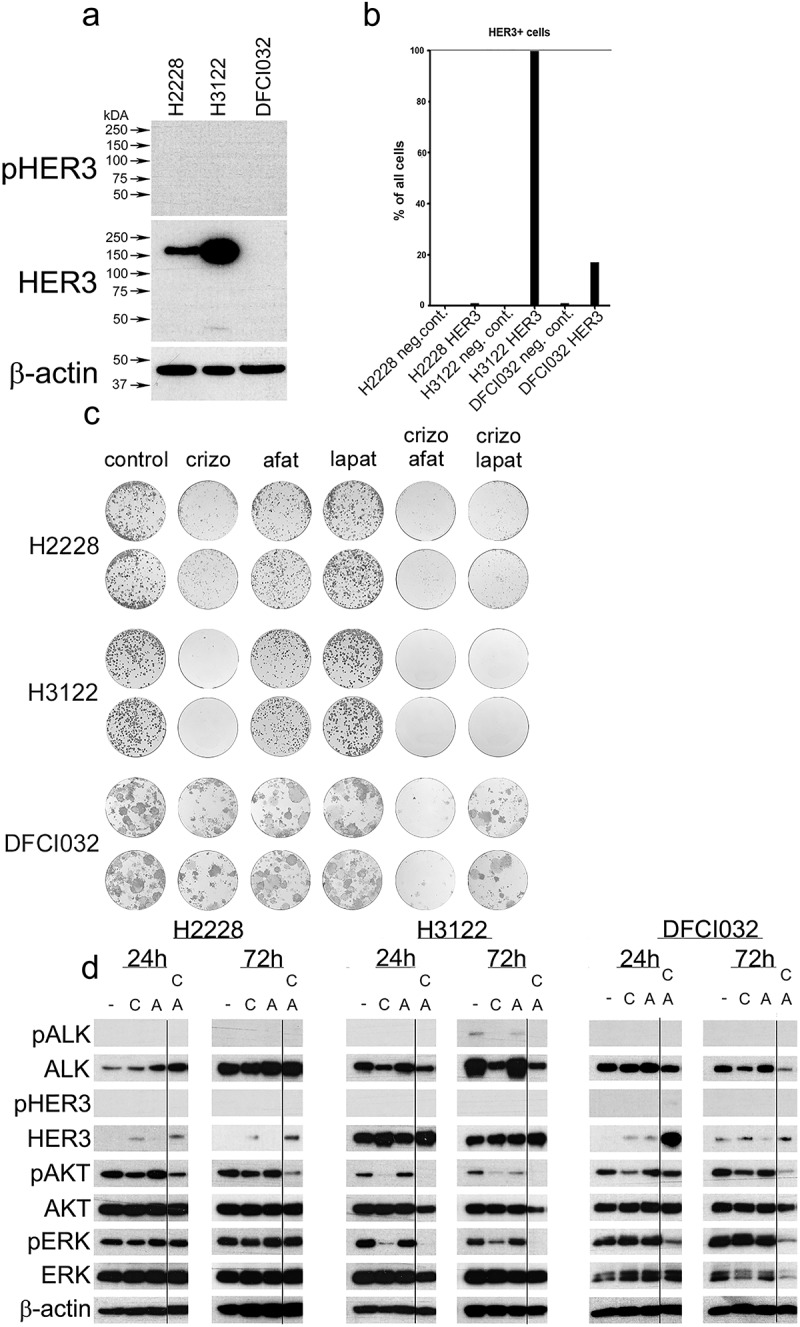
Colony formation and cell signalling in response to ALK TKI, HER TKI, or combined treatment. a. HER3 expression by western blot in H2228, H3122, and DFCI032 cell lines. b results of the flow cytometry analysis of percentages of HER3 expressed cells in neg. control and HER3 antibody treated samples. c. Colony formation assay of ALK translocated cell lines H2228, H3122, and DFCI032 treated 7 d with ALK TKI, afatinib (pan-HER TKI), lapatinib (HER1–2 TKI) or combination of ALK TKI and HER TKIs. d. Western blot analysis for phosphorylated and total ALK, HER3, AKT and ERK in response to ALK TKI (c), afatinib (a), or their combination for 24 h or 72 h.

*ERBB and ALK TKI combinations in ALK translocated cancel cell lines*. Since ERBB receptors have been linked to ALK TKI resistance, we investigated whether HER3 plays a role in ALK TKI resistance. First, we studied the expression of HER3 in *ALK*+ cell lines. Western blot analysis showed that the expression of HER3 was highest in H3122, followed by low expression in H2228, and DCFI032 expressed non-detectable levels of HER3. Phosphorylated HER3 was not detected in any of the studied cell lines (Figure 1A). HER3 expression was analyzed by FACS to detect the cell membrane expression of HER3. Analogous to the western blot analysis, H3122 had the highest levels of expression, whereas the other cell lines had low levels of expression (Figure 1B, Online Resource 1).

Next, we performed a colony formation assay using crizotinib (ALK TKI), lapatinib (EGFR and HER2 TKI), and afatinib (pan-ERBB TKI) in *ALK*+ NSCLC lines. The single-agent crizotinib significantly reduced colony formation in all cell lines, whereas afatinib had a modest effect on H3122 cells. Compared to the single-agent crizotinib, a marked reduction in colony formation was observed in all cell lines treated with crizotinib and afatinib, and this was statistically significant in H2228 and DFCI032 cells. Conversely, a reduction in colony formation with lapatinib combination was observed in H2228 and H3122 cells, but this did not reach statistical significance (Figure 1C, Table 1).

**Table 1.** Colony formation in response to TKI treatments.

Cell line	Treatment	Area of Colonies	SD	p-value*
H2228	Control	35.25	0.35	
	Crizotinib	10,60	0.42	<.001
	Afatinib	18.90	1.70	NS
	Laptinib	29.30	5.23	NS
	Crizotinib	10,60	0.42	
	Crizotinib+Afatinib	3.0	0.28	.02
	Crizotinib+Lapatinib	5.5	0.42	.07
H3122	Control	25.35	3.46	
	Crizotinib	1.1	0.42	.01
	Afatinib	12.60	0.14	.04
	Laptinib	29.50	2.26	NS
	Crizotinib	1.1	0.42	
	Crizotinib+Afatinib	0.25	0.07	NS
	Crizotinib+Lapatinib	0.25	0.07	NS
DFCI032	Control	57.75	5.44	
	Crizotinib	28.90	0.14	.03
	Afatinib	48.30	2.97	NS
	Laptinib	54.85	4.88	NS
	Crizotinib	28.90	0.14	.03
	Crizotinib+Afatinib	2.75	0.49	<.001
	Crizotinib+Lapatinib	26.70	2.83	NS

SD: standard deviation; *T-test.

Western blot analysis was carried out to study the effects of afatinib and its combination with ALK TKI on cell signaling. ALK phosphorylation was detected only in H3122 cells, and phosphorylation was downregulated in response to ALK TKI. Total ALK expression was downregulated with ALK TKI treatment in H3122 cells and with combination treatment in DFCI023 cells. Phosphorylated HER3 was not detected in any of the lines or treatments, whereas the total HER3 expression increased in response to ALK TKI treatment in all the lines, and the highest expression was detected with the combination treatments. The most significant downregulation of phosphorylated AKT was observed with combination treatment in all cell lines. The downregulation of AKT was more prominent with long-term (72 h) than short-term (24 h) treatment (Figure 1D).

### HER3 knockdown in ALK+ NSCLC cell lines

**Figure 2.**
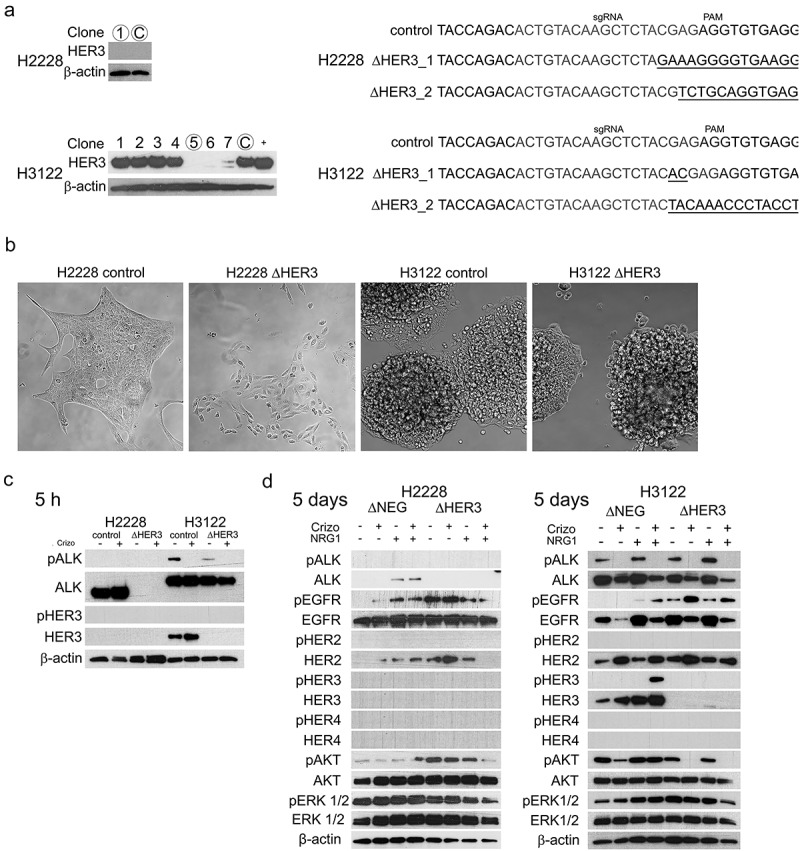
HER3 expression and its knockdown in ALK translocated NSCLC cell lines a. Selected clones for analysis (H2228: 1; H3122: 5) and corresponding knockdown sequences. Clone C is a control. b. Phase-contrast microscopy of the control and knockdown cell lines. c. western blot analysis for phosphorylated and total ALK and HER3 with or without Crizotinib (5 h) treatment. d. western blot analysis for ERBB-family, ALK and their important downstream targets in knockdown H3122 and H2228 (ΔHER3) cell lines and their wild-type counterparts (ΔNEG) treated with either ALK TKI, NGR1 or combination for 5 days.

Based on HER3 expression experiments (Figure 1A), we selected H3122 (high HER3) and H2228 (low HER3) to carry out knockdown experiment for HER3. HER3 expression was downregulated using the CRISPR-Cas9 system. For H2228, we generated only a single HER3 knockdown clone (*n* = 1), which was verified by sequencing. Due to the low levels of HER3 expression in H2228 cells, we could not detect the expression difference in the western blot. In the H3122 cell line, we were able to generate multiple clones with low or undetectable levels of HER3. Clone no. 5 was selected for further analysis, and the knockdown was verified by sequencing (Figure 2A).

Morphological analysis of HER3 knockdown clones revealed that H3122 retained its original morphology, with a clustering-type growth pattern. Surprisingly, the morphology of the H2228 HER3 knockdown line was markedly altered from the original epithelia-sheet-like growth pattern to a spread-out single-cell pattern with a very slow growth rate (Figure 2B). Next, we analyzed HER3 knockdown cell lines for ALK expression. In the H2228 cell line, HER3 knockdown resulted in complete loss of ALK expression. Furthermore, ALK and phosphorylated ALK expression was modestly decreased in H3122 cells with HER3 loss compared to control cells (Figure 2C).

Next, we analyzed cell signaling using western blotting in HER3 knockdown lines. Activation of phosphorylated EGFR was detected in HER3 knockdown lines compared to wild-type controls. HER3 knockdown to inhibit NRG1 mediated HER3 phosphorylation in H3122 cells In H3122 cells, HER3 knockdown markedly downregulated phosphorylated AKT in ALK TKI-treated cells, and this was able to inhibit NRG1 mediated AKT reactivation when compared to wild-type H3122 counterparts. In H2228 cells, combination treatment with ALK TKI and NRG1 resulted in the activation of AKT, while the knockdown line resulted in the inhibition of AKT signaling (Figure 2D).

### Association of HER3 and translocated ALK

**Figure 3.**
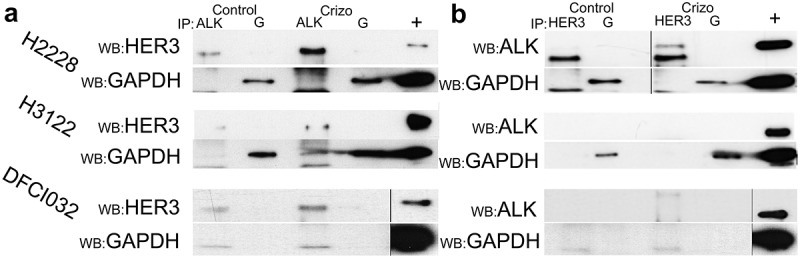
Co-immunoprecipitation of ALK and HER3 in ALK translocated cell lines H2228 and H3122. a. Immunoprecipitation (IP) with ALK antibody and GAPDH (G) antibody and detection with HER3 or GAPDH antibodies. TKI treatment used was 2 h. b. Immunoprecipitation with HER3 antibody and GAPDH antibody and detection with ALK or GAPDH antibodies. + a positive control without immunoprecipitation. Y-axis labels protein sizes in kDa.

Since HER3 knockdown resulted in altered levels of translocated ALK and knockdown was able to markedly reduce downstream signaling of ALK to AKT in H3122 cells, we speculated that these two proteins could interact. We performed co-immunoprecipitation (IP) of ALK and HER3 in the H3122 and H2228 cell lines. When IP was performed using an ALK antibody, HER3 co-immunoprecipitation was detected in ALK TKI-treated cells (Figure 3A). When IP was carried out with the HER3 antibody, ALK co-immunoprecipitation was detected in the H2228 cell line, but not in the other cell lines (Figure 3B).

**Figure 4.**
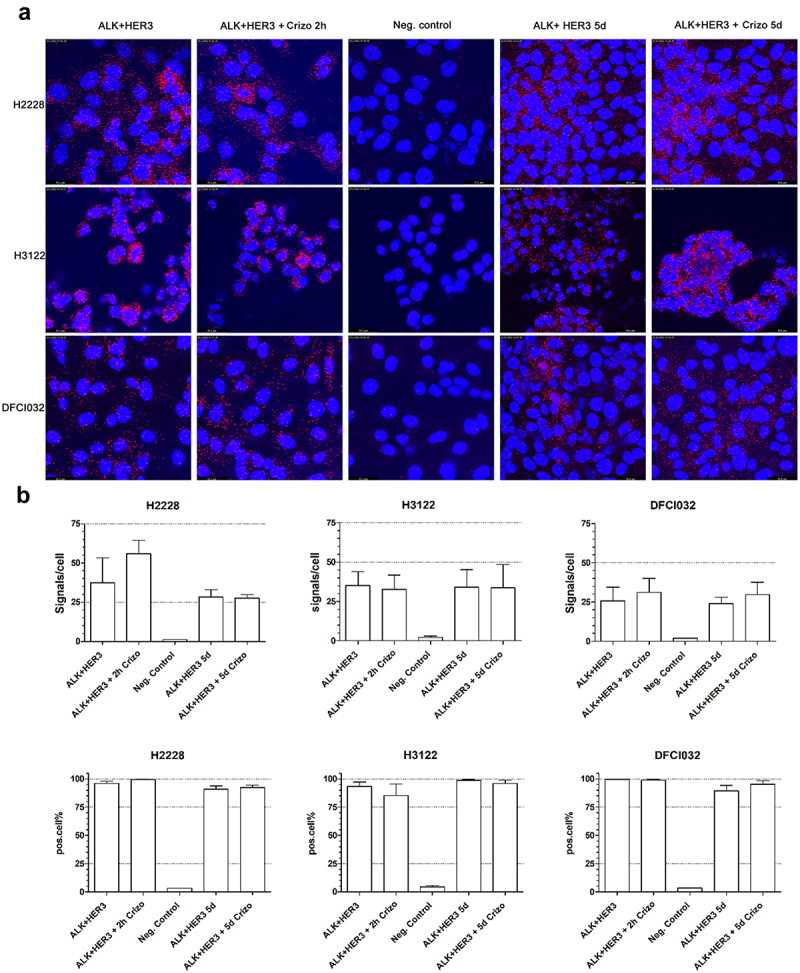
a. Proximation ligation assay for protein–protein interactions of ALK and HER3 in ALK translocated NSCLC cell lines. ALK TKI treatment was applied for 2 h or 5 days. In negative controls, primary antibodies were omitted. b. Proximation ligation assay positive signals per cell in control and TKI treatments and percentages of positive signals.

A proximity ligation assay (PLA) was used to further verify the protein–protein interaction between HER3 and translocated ALK. In PLA experiments, HER3 and ALK protein–protein interactions were verified to be present in all the *ALK* translocated cell lines, and very scarce signals were detected in negative controls. Next, we assessed whether ALK TKI treatment altered the amount of colocalization signal. After 2 h of ALK TKI treatment, we did not detect any changes in the presence or intensity of protein–protein interaction signals. When ALK TKI treatment was applied for 5 days, we visually detected significantly more intense signals in the H3122 and H2228 cell lines, while the differences in the number of cells or signals per cell were not statistically significant (Figure 4, Online Resource 2).

**Figure 5.**
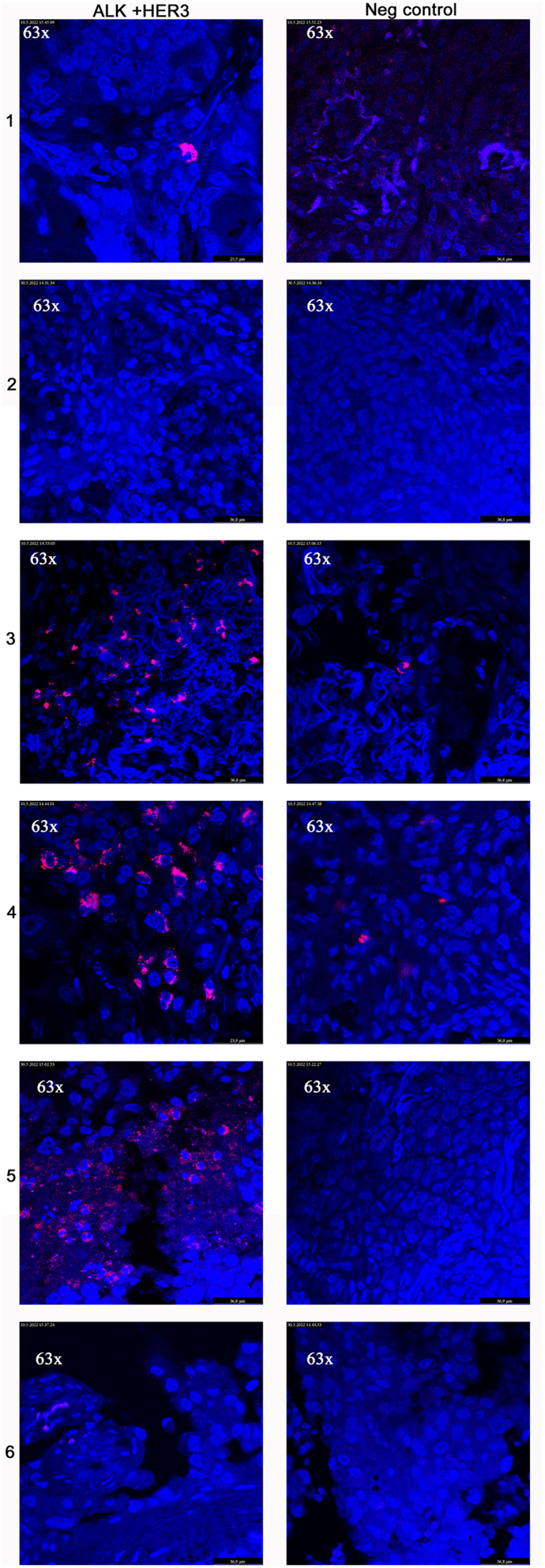
Proximation ligation assay for ALK and HER3 interactive co-expression in ALK translocated NSCLCs (*n*= 6). In negative controls, primary antibodies were omitted.

PLA was used to study the protein–protein interaction of HER3 and ALK in ALK-translocated NSCLC tumors (n = 6). We could detect the protein–protein interaction signals in four of the tumors (66.7%). The signal was altered between the tumors, and some presented it in the vast majority of the tumor cells (n:o 3–5) while in one sample (n = 1), a very intense signal was observed in single cells in the whole tumor (Figure 5). The mean signal for PLA per cell in ALK+ tumors was 17.17 (SD 21.62), while this was 1.33 (SD 2.57) in negative controls (p = .020).

### HER3 expression in ALK translocated NSCLCs

**Figure 6.**
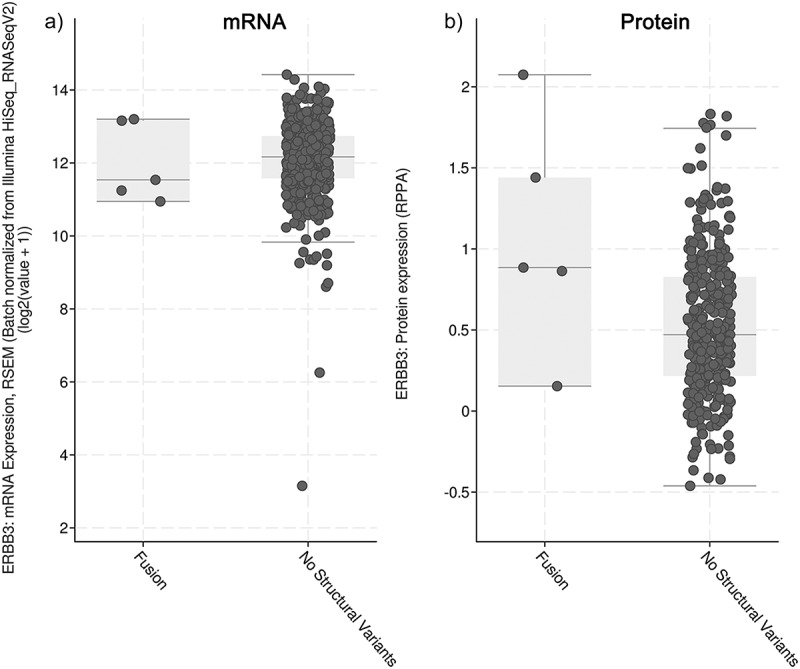
*In silico* analysis for HER3 expression in ALK translocated NSCLC tumors compared to wild-type adenocarcinomas. A. mRNA expression. B. Protein expression.

*In silico* analysis of HER3 and ALK co-expression was performed using Bioportal software. HER3 expression data were only available for five cases of ALK-translocated NSCLC, and these were compared with NSCLC cancers with adenocarcinoma histology. mRNA expression of HER3 in ALK-translocated cancers was similar to that in the wild type, while the median protein expression of HER3 was higher; however, the difference was not statistically significant (Figure 6).

## Discussion

In *ALK* translocated NSCLC, drug resistance plays an important role in disease management in ALK-translocated NSCLC. Novel TKIs have been used to overcome on-target and pharmacokinetic resistance commonly occurring with first-generation TKI crizotinib. However, off-target resistance mediated by the activation of bypass signaling mechanisms is of growing importance. In this study, we investigated HER3 mediated ALK TKI resistance and HER3 targeting as a means of overcoming drug resistance.

ERBB family activation has previously been linked to ALK TKI resistance by multiple investigators. Most studies involve only *in vitro* models, but some studies have also shown this phenomenon to exist in patients.^28^ Of which ERBB-family EGFR, HER2, and HER3 have been shown to mediate ALK TKI resistance. As an example, *ALK+* DFCI032 cell line, which is primary resistant to all ALK TKIs, have concurrent activation EGFR and HER2 co-targeting of EGFR and HER2 with ALK-induced apoptosis in this line.^20^ In the manner HER3 mediated resistance, our results confirmed previous findings. Other described bypass resistance mechanisms include activation of FGFR2&3 and cMET.^29^ Interestingly, previous studies have identified and receptor tyrosine kinases (RTK), such as FGFR2, can act as an upstream activator of ERBB3.^30^ In addition, metabolic reprogramming of 6-phosphofructo-2-kinase/fructose-2,6-biphosphatase 3 (PFKFB3) via STAT3 has recently been linked to ALK TKI resistance.^31^ Clinical evidence of co-targeting multiple signaling pathways is still missing in *ALK* translocated NSCLC. However, many of the characterized resistance mechanisms are generally targetable by pharmacological agents. This includes HER3, which can be pursued with broad-spectrum ERBB TKIs such as afatinib and HER3 targeting antibody drug conjugates. Furthermore, some investigators have provided evidence that co-targeting with histone deacetylase or angiogenesis inhibitors can overcome ALK TKI resistance.^23,32^

The novelty of the current study lies in the potential interaction between translocated *ALK* and HER3. Surprisingly, knockdown of HER3 led to downregulation or loss of expression in translocated ALK, which inhibited downstream signaling to AKT. Furthermore, distinctive morphological transformations in response to HER3 knockdown were observed in one *ALK+* cell line. Based on these findings, we speculated that translocated ALK and HER3 could interact. We provided evidence that this interaction is present *in vitro* by two methods (IP and PLA) and *in vivo* using human ALK-translocated tumor samples. One could speculate that the ALK-HER3 interaction may play a role in TKI resistance, especially in persisting or dormant cells. Even though HER family activation mediated resistance to ALK TKIs have been characterized previously by many, to our knowledge current study is the first to characterize the potential interaction between these two proteins.^20,28^

The findings of our study and those of others have provided evidence that HER3 is an important player in ALK TKI resistance. To our knowledge, there are no ongoing clinical trials investigating the co-targeting of ALK and HER3 in ALK-positive NSCLC. If HER3 plays a role in ALK downstream signaling, the mechanism could mediate both intrinsic and acquired resistance to ALK TKIs. Our work lays the groundwork for potential clinical trials investigating co-targeting in both clinical resistance settings.

Our study had some limitations. We investigated ALK and HER3 interactions using *in vitro* models, which might have limited correlation with *in vivo* settings. An effort was made to investigate potential protein–protein interaction sites by biocomputing. However, this did not reveal any leads because both proteins are very large and 3D structures are available only for some parts of the proteins. Without any biocomputing leads, carrying out further site-modification experiments on target proteins is not feasible.

In conclusion, we showed that HER3 plays a role in ALK TKI resistance and that HER3 co-targeting can increase the therapeutic efficiency of TKIs in ALK-positive NSCLC. More importantly, we characterized the molecular mechanism by which HER3 mediates resistance to direct interactions between translocated ALK and HER3. The results of this study lay the foundation for further preclinical and clinical investigations.

## Supplementary Material

Supplemental Material

## Data Availability

All data generated or analyzed in this study are included in this published article. The raw data used in the analyses are available upon reasonable request.
