# Supplementary material for: Role of human epidermal growth factor receptor 3 in treatment resistance of anaplastic lymphoma kinase translocated non-small cell lung cancer
Source: Cancer Biol Ther. 2023 Sep 18;24(1):2256906. doi: 10.1080/15384047.2023.2256906 (PMC10512822; doi:10.1080/15384047.2023.2256906)
Supplement: Supplemental Material [file KCBT_A_2256906_SM4467.docx]

Article title: ”Role of human epidermal growth factor receptor 3 in treatment resistance of anaplastic lymphoma kinase translocated non-small cell lung cancer”

Authors; Tiia J Honkanen^1,2,3^, Milla E K Luukkainen^1,2,3^, and Jussi P Koivunen^1,2,3^

Affiliations: ^1^Department of Oncology and Radiotherapy, Oulu University Hospital, POB 20, 90029 OYS, Finland

^2^Medical Research Center Oulu, POB 5000, 90014 Oulu, Finland

^3^Cancer and Translational Medicine Research Unit, University of Oulu, POB 5000, 90014 Oulu, Finland

E-mail address of the corresponding author: jussi.koivunen@ppshp.fi Journal name: Cancer Biology and Therapy


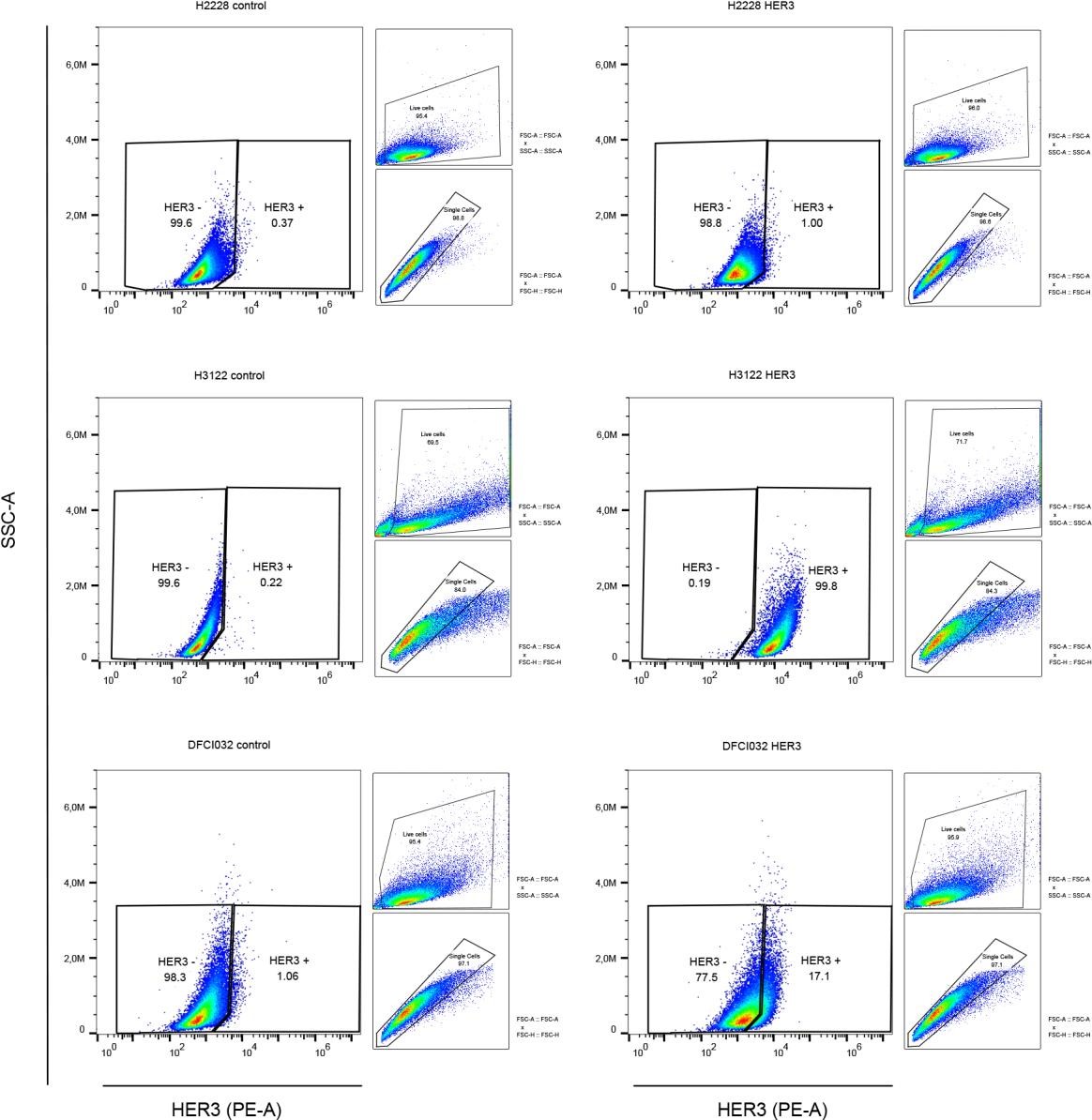


Online Resource 1, supplementary figure1. Used FACS gates in FlowJo analysis of HER3 expression.

**Supplementary Table 1.** Proximation ligation assay for HER3 and ALK protein-protein interaction in ALK+ NSCLC lines.

| H2228 | Control (2h) | 95.91 | 3.58 |  | 37.30 | 27.67 |  |
| --- | --- | --- | --- | --- | --- | --- | --- |
|  | ALK TKI 2h | 99.23 | 1.33 | NS | 55.83 | 15.07 | NS |
|  | Neg control | 1.56 | 2.21 | <0.001 | 0.60 | 0.84 | NS |
|  | Control (5d) | 90.78 | 0.42 |  | 28.21 | 8.08 |  |
|  | ALK TKI 5d | 92.17 | 0.28 | NS | 27.59 | 2.18 | NS |
| H3122 | Controli (2h) | 93.43 | 6.52 |  | 15.54 | 8.97 |  |
|  | ALK TKI 2h | 85.28 | 17.55 | NS | 15.48 | 8.94 | NS |
|  | Neg control | 4.25 | 1.85 | <0.001 | 2.18 | 1.17 | NS |
|  | Controli (5d) | 98.75 | 1.32 |  | 34.00 | 19.41 |  |
|  | ALK TKI 5d | 96.05 | 5.13 | NS | 33.73 | 25.60 | NS |
| DFCI032 | Control (2h) | 99.60 | 0.56 |  | 25.78 | 8.60 |  |
|  | ALK TKI 2h | 98.79 | 1.41 | <0.001 | 31.09 | 9.01 | NS |
|  | Neg control | 1.72 | 2.44 | 0.0 | 25.78 | 12.16 |  |
|  | Control (5d) | 89.41 | 8.46 |  | 23.94 | 6.94 |  |
|  | ALK TKI 5d | 95.31 | 5.15 | NS | 29.73 | 13.58 | NS |

**Cell line Treatment Positive cells (%) SD p-value^*^ Signals/cell SD p-value**

SD= standard deviation; ^*^T-test

Online resource 2
